# Notes and News

**Published:** 1890-10-11

**Authors:** 


### Notes and News.

Collected and Communicated.

Another scandal has occurred at Chester Workhouse; Dr. Downes, of the Local Government Board, found a child suffering from spinal disease, with no dressing on the abscess on her back. Dr. Downes naturally recommends that there be at least one paid night nurse, and that imbeciles be put under the charge of paid officials instead of fellow paupers. The nurse was censured by the Board of Guardians for neglecting the case mentioned above, and the medical officer was called upon to resign. But Dr. Harrison calmly announced he had no intention of resigning and some lively meetings of the guardians are expected.

Dr. Caffyn, of Liquor Carnis fame, adds to his other employments that of novel writing and has lately published a story of a morphiomaniac called "A Poppy's Tears." The story is interesting, full of clever sayings, but carelessly put together, and bears evidence of hasty workmanship. The inaccuracies in the quotations from Browning are most trying to the admirer of that poet. Dr. Caffyn does not write in the least in the orthodox manner; he lets his hero die of morphia, and leaves us to imagine the fate of the heroine. This is rather refreshing; but we should have enjoyed the book much more if a little more skill and labour and thoroughness had been employed on its construction.

A small Convalescent Home for Women was opened at Hurley last week by Dr. Randall, the Suffragan Bishop of Reading. The house has been used for a short period as a Sunday-school for children, but has been appropriated to its present purpose, for which it is well adapted, through the instrumentality of two ladies. The accommodation is good, if small, and the idea is in the first place to help the poor of East and South London, although it will not be entirely confined to London, subscribers of a guinea being able to recommend any patient for three weeks. Intended for people requiring change of air rather than great attention, the home ought to do valuable work. It stands in the midst of beautiful scenery, and has a garden and orchard attached. The institution has been opened free of debt, and several subscriptions are opened for the future.

A correspondent, writing from Colombo, Ceylon, says: "The chaplain of the Leper Hospital here asked me the other day if I could get any of my English friends to send him some pictures for the children—old illustrated papers, scrap books, &c., anything of that kind. I have been to the hospital twice. It is in a beautiful spot and is a fine hospital with lovely grounds. Of course the disease is most distressing, some of the patients are little boys in the first stage only; others have lost toes, fingers, &c." Now that so much interest is taken in the leper question, we are sure some of our readers will respond gladly and fully to the above appeal. Our correspondent is himself a clergyman and a worker in Ceylon.

Hospital Saturday at Cardiff was a memorable day this year, the novel idea of a cyclists' procession with illuminated machines, which appears to be gaining such popularity, having been most successfully carried out. There is some difference in the various estimates of the number of cyclists who actually took part in the function, but, accepting the lowest one of 260, the scene must have been uncommonly effective. Every conceivable device was resorted to in the adornment of the cycles, and many of their riders added to the carnival air by assuming extravagant fancy dresses. All along the route collectors were stationed, and, favoured by fine weather, the lantern parade was an unqualified triumph, the total amount obtained from all sources for the infirmary being £230 15s. 8d.

At Swindon Hospital Saturday is driving Hospital Sunday out of the field. This year the day's proceedings began with a parade of friendly societies, followed by a programme of entertainment at the Lawn, by permission of Mr. Goddard, comprising a dramatic sketch, minstrel performance, a public tea, and dancing. In the late evening there was a monster torchlight procession and masquerade. Conspicuous in the procession were several trolleys equipped as a gas-fitter's shop, shoeing forge, blacksmith's forge, and a hospital, while the local cyclists covered the tyres of their machines with Chinese paper illuminated from inside. All the arrangements appear to have been well carried out, and it is probable that the accounts, when made up, will give a balance in favour of the hospital of over £100.

A perfectly astounding story of mismanagement in connection with the fever hospital at Portobello is published by the *Edinburgh Evening Dispatch*. In the first place the demand for a fever hospital at all was strenuously resisted, and after its necessity had been admitted time was again frittered away in squabbling over the size it should be. When it was finally built, a committee of three undertook the furnishing, actually proposing carpets, stuffed mattresses, and cretonne-covered stuffed easy chairs. Though some of this folly was prevented, the chairs have somehow been introduced. Before the place was furnished a case of scarlatina occurred, and, after some negotiation, was admitted to the hospital. A bed having been obtained by the Sanitary Inspector, the mother and child were left to shift for themselves, no nurse having been appointed, and no provision made for the supply of medicine and food. After having somehow saved her child's life and gone home to Glasgow, the mother received a bill from the Portobello authorities for £4 "for accommodation." She rendered a contra account for £4 7s. for nursing, food, medicine, and "carbolic soap for washing the doctor's hands," and laid the matter before the high medical officer of Glasgow, and through him to the Board of Supervision. The latter applied for an explanation to the Portobello Hospital Board, and this explanation we await with interest. The hospital is now under the charge of a lamplighter and his wife, and in it there is a second case of scarlatina, she also being nursed by her mother.

THE following vacancies are announced this week, the amount of the salary in each case being given after the name of the institution:—

**Resident Medical Officers.**

Evelina Hospital for Children, House Surgeon—£70, board, etc.
North-West London Hospital, Assistant Medical Officer—board, etc.
North-West London Hospital, Medical Officer—£50, board, etc.
Paddington Green Children's Hospital, House Surgeon—£50, board, etc.
East London Hospital for Children, House Physician—board, etc.
Whitechapel Union Infirmary, Assistant Medical Officer—£150, board, etc.
Lincoln County Hospital, House Surgeon—£100, board, etc.
Flintshire Dispensary, House Surgeon—£120, rooms, etc.
West Riding Asylum, Fourth Assistant Medical Officer—£100, board, etc.
Birmingham City Asylum, Assistant Medical Officer—£120, board, etc.
Cardiff Union, Assistant Medical Officer—£100, board, etc.
Leeds General Infirmary, Resident Obstetric Officer, two House Physicians, two House Surgeons, one Resident Officer—board, etc.

**Non-Resident Medical Officers.**

Taunton and Somerset Hospital, Hon. Physician.
Nottingham General Hospital, Hon. Physician.
Parish of Portree, Medical Officer—£71.
Parish of Shapensay, Medical Officer—£80.

MRS. ADAM CLARKE opened the hospital at Longton on September 25th. It was felt peculiarly fitting that Mrs. Clarke should perform the opening ceremony, as half a century ago the old hospital was founded by her husband. This developed into the hospital at Mount Pleasant, the land for which was given by the late Mr. Draycott in 1866. But the population of the town has increased so rapidly that a larger and more commodious hospital has been long a necessity. Sir Smith Child, the leading philanthropist of the district, took the matter in hand, and the Mayor of Longton (who has held that position for four years) organised a "supply fair" in Longton market for the cause. This was a magnificent success. The Mayor and his committee begged all the produce, and £1,150 was the result. This was supplemented by large contributions from the local residents, and £4,500 has been subscribed. The new hospital is a handsome building, land has cost £6,000 in erection.

THE Hope Hospital scandal having ended in the demand for the resignations of Dr. Conry and Dr. Walker, and the accepting of the resignation of Nurse Humphreys, there was some surprise at the meeting of October 3rd at neither of the medical men sending in the required resignations; indeed, Dr. Conry wrote refusing to resign. Mr. J. H. Bowden moved a resolution that the Local Government Board having directed Dr. Conry to send in his resignation and Dr. Conry having failed to do so, the Board hereby dismiss Dr. Conry from his office. The Local Government Board had done their part in regard to the inquiry, and Dr. Conry had treated their communication in a contumacious spirit. The Hope Hospital administration needed putting on proper ground. Mr. Mottram thought Dr. Conry had been very harshly treated, as it seemed that the inquiry had, so far as the charges went, resulted in his favour. He thought that the Local Government Board should be informed of what the Guardians had already done in the matter, and that it should be left to them to deal with as they thought fit. Mr. Bowden, replying, said the hospital was suffering all the while the matter was going on. Only last week the committee had to deal with complaints against other people there, and while they were considering how to make a full and complete change the patients were suffering. The amendment was put and lost, and the original resolution of Mr. Bowden, dismissing Dr. Conry was then carried. Mr. Worsley moved a similar resolution dismissing Dr. Walker. No reply had, he said, been received from that gentleman in regard to the previous communication of the Guardians, but they were determined that the organization of the hospital should rest on a new basis.

IN case any of our readers have missed, in the course of their holidays, our notice about the gifts for the adult wards of the metropolitan hospitals at Christmas, we beg to repeat that we hope busy fingers are making us shawls, socks, petticoats, etc., to distribute at the festive season. It is not such interesting work to sew or knit for adults as for children, but if our readers had seen the wistful faces of the elder convalescents at a hospital Christmas tree, they would understand why we make this request. The children are already well cared for at Christmas; may the adults also be so this year. We have to acknowledge from L. W., two comforters, three aprons, three pair of knee-caps, and four pair of mittens; and from E. W. B. a parcel of men's clothing.

> "The only garments that endure
> Are kindly gifts to clothe the poor;
> For neither tatter, time, nor moth
> Shall fray that silk or fret that cloth."

THE first half-yearly meeting of the Bristol Royal Infirmary has been held, the quarterly meetings having been done away with. The Chairman said that since their last meeting the night superintendent, Mrs. Mathias, had resigned, and her position had been taken by Mrs. Robson. The Camden House, which they took for the nurses, was going on extremely well. It was an important addition to the infirmary and an addition to their expenditure, but by-and-bye he hoped it would not be so great an addition to their expenditure. Since he had been a member of the committee of the institution the expenditure had risen two thousand a-year. He believed when he first joined the committee, twenty-two years ago, the expenditure was about £10,000 a-year, but now they could hardly reduce it below £12,000. They needed more subscriptions from the residents of the neighbourhood and city.

CONSUMPTION hospitals are institutions the value of which can hardly be over-estimated, and it will be well if the advocates of such an institution for Belfast are successful in carrying their idea into execution. The average annual death-rate from consumption in Belfast for the ten years ending 1880 was 38·2 per 10,000 persons; in North Dublin, 33·4; and in Newtownards, 31·7. In Belmullet the death-rate was only 4·8, from which it might be inferred that if the conditions of life in Belfast could be put on the same footing as they are in Belmullet, 34 persons in every 10,000 or 850 persons every year would be saved from dying of consumption. Although this, of course, is practically impossible, there is, no doubt, but that a hospital for consumption is very much wanted in Belfast to counteract as far as may be the climatic and atmospheric peculiarities which are so favourable to the disease, and it is much to be hoped that the initial pecuniary difficulties that at present block the scheme will before long be removed.

THE opening of the Cottage Hospital at Thirsk by Mrs. Lambert, of Sowerby, was an imposing function; all the county people were present. The hospital is a pretty little building, from designs by Mr. J. M. Bottomley. Over the main entrance is a carved panel with shield bearing the Lambert coat of arms, and on the keystone to the arch over the doorway is a carved ribbon bearing the inscription, "The Lambert Memorial Hospital." Carved bosses to terminate the label mould over the doorways and windows, and the ball terminals to each gable, give to the appearance of the building a very pleasing effect. The roofs are covered with red Staffordshire tiles. The principal part of the building is occupied by two large wards, with nurses' room between, each of the wards having access to a verandah, and the surgery, accident, or occasional ward. These rooms and other portions of the hospital have been furnished in the most suitable manner, no expense being spared to make the hospital an efficient one.

